# The “Blood pressure and oxygenation targets in post resuscitation care, a randomized clinical trial”: design and statistical analysis plan

**DOI:** 10.1186/s13063-022-06101-6

**Published:** 2022-02-24

**Authors:** Jesper Kjaergaard, Henrik Schmidt, Jacob E. Møller, Christian Hassager

**Affiliations:** 1 Department of Cardiology, The Heart Centre, Copenhagen University Hospital Rigshospitalet, Copenhagen, Denmark; 2 Department of Clinical Medicine, University of Copenhagen, Copenhagen, Denmark; 3 Department of Cardiothoracic Intensive Care, Odense University Hospital, Odense, Denmark

**Keywords:** Cardiac arrest, Targeted temperature management, Blood pressure targets, Oxygenation targets, Post-cardiac arrest care

## Abstract

**Background:**

Comatose patients admitted after resuscitation from cardiac arrest have a significant risk of poor outcome due to hypoxic brain injury. While numerous studies have investigated and challenged the target temperature as the efficacious part of the guideline endorsed Targeted Temperature Management (TTM) protocols, our knowledge and how the remaining parts of the TTM are optimized remain sparse. The present randomized trial investigated two aspects of the TTM protocol: target blood pressure during the ICU stay and oxygenation during mechanical ventilation. Furthermore, the efficacy of device-based post-TTM fever management is addressed.

**Methods:**

Investigator-initiated, dual-center, randomized clinical trial in comatose OHCA patients admitted to an intensive cardiac care unit. Patients are eligible for inclusion if unconscious, older than 18 years of age, and have return of spontaneous circulation for more than 20 min.

Intervention: allocation 1:1:1:1 into a group defined by (a) blood pressure targets in double-blind intervention targeting a mean arterial blood pressure of 63 or 77 mmHg and (b) restrictive (9–10 kPa) or liberal (13–14 kPa) of arterial oxygen concentration during mechanical ventilation. As a subordinate intervention, device-based active fever management is discontinued after 36 h or 72. Patients will otherwise receive protocolized standard of care according to international guidelines, including targeted temperature management at 36 °C for 24 h, sedation with fentanyl and propofol, and multimodal neuro-prognostication. Primary endpoint: Discharge from hospital in poor neurological status (Cerebral Performance category 3 or 4) or death, whichever comes first. Secondary outcomes: Time to initiation of renal replacement therapy or death, neuron-specific enolase (NSE) level at 48 h, MOCA score at day 90, Modified Ranking Scale (mRS) and CPC at 3 months, NT-pro-BNP at 90 days, eGFR and LVEF at 90 days, daily cumulated vasopressor requirement during ICU stay, and need for a combination of vasopressors and inotropic agents or mechanical circulatory support.

**Discussion:**

We hypothesize that low or high target blood pressure and restrictive and liberal oxygen administration will have an impact on mortality by reducing the risk and degree of hypoxic brain injury. This will be assessment neurological outcome and biochemical and neuropsychological testing after 90 days.

**Trial registration:**

ClinicalTrials.gov NCT03141099. Registered on May 2017 (retrospectively registered)

## Administrative information

‘Note: the numbers in curly brackets in this protocol refer to SPIRIT checklist item numbers. The order of the items has been modified to group similar items (see http://www.equator-network.org/reporting-guidelines/spirit-2727-statement-defining-standard-protocol-items-for-clinical-trials/).

**Table:** 

*Title {1}:*	Blood pressure and oxygenation targets in post resuscitation care, a randomized clinical trial
*Trial Registration {2a and 2b}*	Clinical trials identifier: NCT03141099 Ethics Committee approval: no. H-16033436
*Protocol version {3}:*	Version 3.2 (07JAN2018)
*Funding {4}:*	NovoNordisk Foundation: NNF17OC0028706. DKK 7,573,000 for the full trial budget
*Author details {5a}:*	Department of Cardiology, The Heart Centre, Copenhagen University Hospital Rigshospitalet, Copenhagen, Denmark (JK, CH, JEM) Department of Cardiothoracic Intensive Care, Odense University Hospital (HS, JEM)
*Name and Contact information for the trial sponsor {5b}*	Jesper Kjaergaard, consultant, MD, PhD, DMScDepartment of Cardiology, The Heart Centre, Copenhagen University Hospital Rigshospitalet, Copenhagen, Denmark. Jesper.kjaergaard.05@regionh.dk
*Role of Sponsor {5c}:*	This is a sponsor-investigator-initiated study with no funding or involvement from pharmaceutical companies, and the sponsor-investigator maintains authority over all aspects of the trial including, design, management, interpretation of results, and publication.

## Introduction

### Background and rationale {6a}

In Europe, 19–104 patients per 100,000 inhabitants per year suffer from out-of-hospital cardiac arrest and resuscitation is attempted [1]. Mortality after OHCA is high, and for patients hospitalized alive, survival has been reported to vary between 34 and 56% [2–5]. Furthermore, the frequency of persistent neurological deficits varies considerably [2, 5–9].

Less than 10% survives out-of-hospital cardiac (OHCA) arrest [10, 11], the in-hospital survival is reported at 30–50% [12, 13], and with neurological injuries remain the leading cause of death [14]. The brain of a patient resuscitated after cardiac arrest (CA) may have been exposed to ischemia and when spontaneous circulation is re-established, the subsequent reperfusion may cause further damage [15]. Thus, both brain ischemia and reperfusion injury lead to tissue degeneration and loss of neurological function, where the extent depends mainly on duration of the no-flow and low-flow periods. Temperature control and Targeted Temperature Management (TTM) targeting 33–36 °C may mitigate this damage and are recommended in current international guidelines [16–20], and recently, avoiding fever may have similar efficacy as actively cooling to 33 °C [21]. However, managing post-cardiac arrest patients is much more complicated than focusing on temperature alone, and maintaining adequate blood pressure and oxygenation as part of post-resuscitation care is emphasized [9;11]. However, targets for managing hemodynamics and mechanical ventilation remain to be firmly supported by prospective randomized trials.

The current study investigates two goals in the guideline-based critical care of comatose patients resuscitated from OHCA: target mean arterial blood pressure and arterial oxygen concentration.

#### Blood pressure targets

While the American and European guidelines agree that MAP below 65 mmHg should be avoided [19, 20], there is no evidence supporting targeting blood pressures higher than 65 mmHg. Two recent controlled but unblinded trials using surrogate endpoints as markers of degree of brain injury, namely MRI [22] and neuron-specific enolase [23], did not show significant benefit of targeting higher blood pressure levels, but were underpowered for showing an effect on clinically relevant outcomes. However, a subsequent post hoc analysis suggested lower concentrations of a marker of brain injury, namely neurofilament light chain in the high-MAP group [24]. Registry-based analyses on a larger number of patients show conflicting results, but suggest that hypotension should be avoided [25–27]. Vasopressors are needed in the vast majority of resuscitated comatose OHCA patients to keep MAP above 65 mmHg [28], with the potential adverse effects of vasopressor therapy.

The important question for clinicians is how to decide on the optimal MAP after cardiac arrest. A clinical equipoise remains, and while considerable effort is put into managing comatose patients after cardiac arrest, large studies of MAP modification with noradrenaline, a cheap and easily adopted intervention, are needed, as guidelines are largely based on expert consensus [29].

#### PaO_2_ targets

The guidelines recommend that oxygen saturations of 94–98% are targeted in the post-resuscitation period [30]. Recently, a target of 10–13 kPa (75–100 mmHg) has been added to the European guidelines [20]. Oxygenation has also been the target of 1 prospective trial, failing to show any benefit of the high target outside the normal range on neuron-specific enolase levels as a surrogate marker of anoxic brain injury [31]. Oxygenation following acute myocardial infarction, the cause of most cardiac arrests eligible for inclusion in the present trial, did not reduce mortality or heart failure incidence [32, 33], nor did high levels of oxygen reduce mortality in respiratory failure in the critical care unit [34]. A better outcome after a conservatory oxygen strategy was, however, indicated in the subgroup admitted with suspected hypoxic-ischemic encephalopathy in the OXY-ICU trial [35].

The normal target PaO_2_ is usually covering this range by targeting 9–14 kPa during TTM and when mechanical ventilation is needed. The trial will compare two strategies of targeting low-normal PaO_2_ of 9–10 kPa (restrictive) and high-normal PaO_2_ of 13–14 kPa (liberal). Measures and interventions to maintain the allocated PaO_2_ are left at the discretion of the attending physician, but a charter for treatment and maintenance of the allocated PaO_2_ targets is developed as an aid for the attending clinicians.

#### Substudies

A number of predefined substudies have been included as part of the trial and will be described in detail elsewhere. The trial steering group agrees to allow the patients to be included in other interventional trials, if deemed unlike to impact on the efficacy of the blood pressure and oxygenation target interventions.

#### Post-TTM fever management

Post-hypothermia fever is associated with worse clinical outcomes in comatose patients admitted after resuscitation from OHCA [36, 37]. Current guidelines suggest that fever should be avoided after rewarming from TTM [19, 20]. While limited data on the efficacy of actively avoiding fever post-TTM is available, post-TTM fever management was part of the TTM trial [38] as well as the recent TTM2 trial intervention [21]. It has not been tested in clinical trials whether fever is a marker of significant neurological injury only, or whether fever is a target for treatment associated with improved outcome. Furthermore, it is unknown whether active fever control is associated with improved outcome compared to more conservative measures such as paracetamol and uncovering, fans, etc. At admittance to the hospital, the body temperature of resuscitated OHCA patients is reported to be approximately 36 °C [36, 39], but it is then often gradually rising. Elevated body temperature is common during the first 48 h after CA [40, 41] and is associated with worse outcome [36], also following TTM [37]. The post-resuscitation period was previously regarded as the missing link in the chain of survival, but during the last years, this has changed [3]. Research has focused on the use of temperature control with TTM, but the general care of CA patients has improved with standardized active intensive care [42] and attention to coronary reperfusion and circulatory support [43, 44]. No difference in TTM at 33 °C compared to 36 °C was found [38], and recently, TTM targeting 33 °C had similar outcome to a strategy of normothermia and avoiding fever [21].

As a subordinate study, the patients included in the present study will all be randomized 1:1 to be actively fever controlled for 72 h post-OHCA or to have the active fever control device withdrawn at 36 h. Randomization will occur at the time of randomization for the other main treatment allocation strata. Apart from using a mechanical fever control device, management of fever will be at the discretion of the attending physician according to local guidelines. Temperatures and administration of paracetamol and duration of active device-based fever control will be monitored. Fever above 37.5 °C should be managed in both groups for the first 72 h or until the patients are alert and responsive, and fever 38.5 °C should be avoided. Endpoints of this intervention are the same as for the BP and PaO_2_ interventions; analyses will not be performed until these two interventions are unblinded.

#### Advanced hemodynamics after cardiac arrest substudy

A pulmonary artery catheter will be placed at the time of placing the central venous line. Measurement of pulmonary artery pressure, wedge pressure, central venous pressures, and mean systemic arterial pressure (as read on the monitor, blinded to the investigator) will be obtained, as well as cardiac output by thermodilution technique by rapid injection of 10 ml of cold isotonic glucose. Measurements of cardiac output will be performed in triplicate and will be repeated until three measurements with a variation of less than 10% are available.

The hypothesis investigated will be that the higher blood pressure group will have lower perfusion (cardiac output) than the low blood pressure target group. Further secondary outcomes are regarding the impact on systemic and pulmonary circulation, as well as the impacts of the two oxygenation targets on the pulmonary circulation in particular. Furthermore, the impact of demographic and clinical factors on central hemodynamics will be investigated.

#### Automated pupillometry substudy

Automated pupillometry has recently been included in the American as well as in the European guidelines on prognostication after out-of-hospital cardiac arrest [19, 20]. While no recommendation for specific cutoff or which modality of the pupillary assessment is given, some prospective data are available [45, 46], suggesting that independent prognostic information can be obtained from automated pupillometry in these patients.

Automated pupillometry will be performed serially during and after TTM treatment by the Neuroptics® NPI-200 device. Measurement will be stored in the SmartGuard® device (a single-use chin guard or spacer) developed for the device. The data from the automated pupillary assessment can be requested by the clinician, but is not routinely recoded in the clinical charts, as automated pupillometry was not a guideline supported part of the prognostication when the trials were initiated. Automated pupillometry has recently been included in the European and the American guideline, still with no consensus on cutoff values for predicting poor outcome.

The substudy investigates the prognostic significance of automated pupillary assessment as part of the multidisciplinary prognostic assessment of patient resuscitation from cardiac arrest. Previous studies have suggested different cutoffs for poor prognostic outcome and more data from prospective trials.

#### Biobank

A biobank containing blood drawn as several time points has been established. Neuron-specific enolase is the only predefined defined marker of outcome in the main trial, but since the initiation of the trial, several other markers have been investigated and the trial biobank will contribute to further consolidating these findings [47], as well supporting studies on new markers of potential prognostic importance.

#### Cognitive outcome and neurological outcome after cardiac arrest

Impact of the two trial interventions on the cognitive function was estimated by the Cerebral Performance Category score [48, 49], the modified Rankin Scale [50, 51], and the Montreal Cognitive Assessment (MoCA) tool [52, 53].

Furthermore, the quality of life of the patient and next of kin, the IQ-code CA [54], and two simple questions are collected.

#### Brain lactate/pyruvate balance during TTM

By means of microdialysis, via a retrograde central venous line in the jugular vein sampling blood at the jugular bulb, lactate and pyruvate concentrations are measured during the TTM phase in a subgroup (*n* = 60). The protocol for this intervention has been published [55].

### Objectives {7}

### Primary objective

To evaluate the superiority of one of the two blood pressure targets investigated: approximately 63 mmHg and approximately 77 mmHg and, separately, superiority of on the two PaO_2_ targets (restrictive vs. liberal oxygenation) with regard to a combined endpoint of time to:

1. Death from any cause or
2. Discharge from hospital in a state of Cerebral Performance category 3 or 4, whichever occurs first

The hospital stay includes tertiary center as well as the local hospital and or specialized hospital-based neurological rehabilitation.

A stratified analysis by the following predefined design variables will also be performed: sex, age above median, pre-arrest renal failure GFR (< 30 ml/min or current renal replacement therapy), pre-arrest hypertension, pre-arrest chronic obstructive pulmonary disease, shockable primary rhythm, and suspected STEMI on initial ECG.

#### Secondary objective

Analysis will compare the two target blood pressure groups with respect to:

1. Time to
  - Initiation of renal replacement therapy
  - Death
2. Neuron-specific enolase (NSE) level at 48 h
3. MOCA score at 3 months (lowest score allocated to patients not available for follow-up, including deceased patients)
4. Modified Ranking Scale (mRS) and CPC at 3 months
5. NT-pro-BNP at 3 months (highest value allocated to patients not available for follow-up)
6. eGFR and LVEF at last available measurement within 3 months of follow-up
7. Daily cumulated vasopressor requirement during ICU stay and need for combination of vasopressors and inotropic agents or mechanical circulatory support

#### Hypotheses

Blood pressure of approximately 63 mmHg or 77 mmHg does not have similar efficacy in preventing death or serious anoxic brain injury in comatose survivors of out-of-hospital cardiac arrest.

Restrictive (9–10 kPa or 68–75 mmHg) vs. liberal (13–14 kPa or 98–105 mmHg) do not have similar efficacy in preventing death or serious anoxic brain injury in comatose survivors of out-of-hospital cardiac arrest.

### Trial design {8}

Multicenter, randomized trial in 2*2 factorial design allocating comatose OHCA patients to one of two target blood pressures (double blind) and restrictive vs. liberal oxygenation (open label) during ICU stay with blinded outcome evaluation. Both interventions allocate patients in a 1:1 ratio. The design is a superiority trial.

### Methods: participant, interventions, and outcomes

#### Study setting {9}

Patients are included in the trial from the Department of Cardiology, Copenhagen University Hospital, Rigshospital, Copenhagen, Denmark, and Department of Cardiothoracic Intensive Care, Odense University Hospital, Denmark. The participating centers provide highly specialized cardiology, including out-of-hospital cardiac arrest care, invasive coronary revascularization, and advanced mechanical circulatory support for approximately 3.9 million people.

### Eligibility criteria {10}

#### Inclusion criteria

1. Age ≥18 years
2. OHCA of presumed cardiac cause
3. Sustained ROSC^#^
4. Unconsciousness (GCS < 8) (patients not able to obey verbal commands) after sustained ROSC

^#^Sustained ROSC: Sustained ROSC is when chest compressions have been not required for 20 consecutive minutes and signs of circulation persist.

#### Exclusion criteria

1. Conscious patients (obeying verbal commands)
2. Females of childbearing potential (unless a negative HCG test can rule out pregnancy within the inclusion window)
3. In-hospital cardiac arrest (IHCA)
4. OHCA of presumed non-cardiac cause, e.g., after trauma or dissection/rupture of major artery OR cardiac arrest caused by initial hypoxia (i.e., drowning, suffocation, hanging)
5. Known bleeding diathesis (medically induced coagulopathy (e.g., warfarin, NOAC, clopidogrel) does not exclude the patient)
6. Suspected or confirmed acute intracranial bleeding
7. Suspected or confirmed acute stroke
8. Unwitnessed asystole
9. Known limitations in therapy and Do Not Resuscitate-order
10. Known disease making 180 days survival unlikely
11. Known pre-arrest CPC 3 or 4
12. > 4 h (240 min) from ROSC to screening
13. Systolic blood pressure < 80 mmHg despite fluid loading/vasopressor and/or inotropic medication/intra-aortic balloon pump/axial flow device^#^
14. Temperature on admission < 30 °C

#If the systolic blood pressure (SBP) is recovering during the inclusion window (220 min), the patient can be included.

### Who will take informed consent {26a}

Danish legislation permits immediate inclusion of patients in non-pharmaceutical trials and mandates that consent is obtained at first given opportunity following inclusion in the trial.

Consent for inclusion of patients unable to provide informed consent (comatose out-of-hospital cardiac arrest survivors) was obtained from a legal representative, usually a relative, and a medical doctor with expertise in the clinical area, but with no relation to the trial. The latter is obtained via a dedicated SMS service, approved by the ethics committee. The consent from the legal representative or relative is obtained by a medical doctor of the department at the first given opportunity. The ethics committee recommendations of time available and allowing time for considering the consent are being obeyed. Informed consent from the patient is obtained by the same staff once the patient regains consciousness.

### Additional consent provisions for collection and use of participant data and biological specimens {26b}

The consent covers storage, handling, and future non-genetic testing of the biobank material; data storage and access to registry and administrative health care data; and the additional intervention described in the substudies section above (including pulmonary artery catheter, automated pupillometry, retrograde jugular bulb catheter (Odense site only), and follow-up visits and questionnaires).

### Interventions

#### Explanation for the choice of comparators {6b}

As both main interventions (arterial blood pressure and oxygenation targets) are compliant with the current guideline for post-cardiac arrest management, there is no formal hypothesis suggesting the superiority of either intervention. Two-sided tests were applied in the sample size estimation.

#### Intervention description {11a}

##### The blood pressure intervention

The blood pressure target allocation is blinded to caregivers as the blood pressure module (HP/Philips M1006B Invasive Pressure module) will be used for the double-blinded intervention. In half of the modules, the calibration factor will be adjusted in order to have the blood pressure measurements being shown approximately 10 mmHg lower than the patients’ actual blood pressure at 100 mmHg, and in half of the patients, the blood pressure measurements will be shown to be approximately 10 mmHg higher at 100 mmHg. Recent validation and feasibility studies in clinical cases suggested high validity of the method [56]. Targeting 70 mmHg during treatment in both groups will mean an intended blinded comparison of 63 mmHg and 77 mmHg but may prove to be 1–2 mmHg lower than intended, both within the usually acceptable ranges of blood pressure in critically ill patients.

These adjustments will be performed centrally (Core lab at the technical department, Rigshospitalet); the BP modules will be clearly marked and shipped to sites. At the time of randomization, and before the first invasive blood pressure is measured following the patient’s arrival to the ICU, the study BP modules are fitted in the monitor, enabling blinding of blood pressure target allocation to the patient, relatives, and care takers. Non-invasive blood pressure measurement is not allowed unless the patients’ safety is considered to be at risk.

Other invasive blood pressure measurements (i.e., CVP or PAP, LAP) will be performed unblinded.

After invasive blood pressure monitoring is discontinued at the ICU, the modules are shipped back to the core lab, where the calibration is re-read, before the settings are adjusted for the next batch of patients.

##### Oxygen intervention

Serial blood gas measurements will be performed targeting either restrictive (9–10 kPa) or liberal (13–14 kPa) oxygenation during mechanical ventilation. The FiO_2_ is set at 60% initially in the liberal oxygenation group and 30% in the restrictive oxygenation group. FiO_2_ is adjusted to the allocated target but must be increased if PaSat% by peripheral pulse oximetry falls below 93%. Blood gas measurements can be performed as often as deemed necessary. Blood gas analyses are mandated every 2 h for the first 12 h and then every 6 h.

PEEP and other ventilatory settings are left at the discretion of the treating physician. Normocapnia (a target of 4.5–6.0 kPa of PaCO_2_) is suggested as the default goal.

#### Criteria for discontinuing or modifying allocated interventions {11b}

##### Blood pressure targets

The patients’ participation in the blinded blood pressure intervention may be discontinued at any time by the attending physician if the patients’ condition requires knowledge of the true arterial blood pressure value. This is achieved by switching the blood pressure measuring module to a non-study-related module. The patient cannot be re-included in the intervention.

##### Oxygenation target

The patient’s target will be set as the goal in all patients. If the patient is unable to achieve the allocated oxygen target by manipulating FiO_2_ and PEEP or by supine ventilation or bronchiolar lavage, the highest possible values are accepted. Extra-corporeal membrane oxygenation is not considered a treatment option to achieve the study-specific PaO_2_ in the trial, while the treating physician may consider upscaling for clinical reasons. If the patients do not meet formal criteria for extubation due to the allocated target oxygenation, extubation may still be performed at the discretion of the treating physician.

##### Strategies to improve adherence to interventions {11c}

All physicians and nursing staff involved in the care of these patients have been instructed in the intervention targets and trained in the use of the standard operating procedure developed for assisting the staff in achieving the goals the patients are allocated to. The study steering group remains available for advice if needed by the treating physician.

##### Relevant concomitant care permitted or prohibited during the trial {11d}

The trial will allow standardized temperature management in the two groups; however, sites are required to adhere to the same protocol for temperature management in the two groups throughout the study period.

Patients will be mechanically ventilated, sedated (propofol/fentanyl), and when necessary paralyzed with neuromuscular blocking agents to reduce shivering and subsequent heat generation and energy consumption. The core body temperature will be set as quickly as possible at the predefined target temperature, according to intervention allocation, with 4 °C intravenous solutions [57], and commercially available cooling devices [58] at the discretion of the treating physician. The target core temperature is then maintained for 24 h. After the maintenance period, core temperature is gradually raised to normothermia of 37 °C with a rewarming rate of no more than 0.5 °C/h. Depending on the randomization in the subordinate, post-TTM fever trial body temperature will be maintained at normothermia 37±0.5 °C for as long the as the patient is comatose until 72 h by a cooling device or just avoiding post-TTM fever by pharmacological and conservative interventions.

##### Provision for post-trial care {30}

Post-trial care is provided as part of the clinical standard protocols. The intervention ends by discontinuing the arterial line for invasive blood pressure monitoring and extubation, respectively. From that time on, the only study-specific intervention will be the 3-month follow-up visit and questionnaire, for which the patients are invited at the time of hospital discharge.

### Outcomes {12}

#### Primary endpoint

1. Death from any cause or
2. Discharge from hospital in a state of Cerebral Performance category 3 or 4, whichever occurs first

#### Secondary objective

Analysis will compare the two target blood pressure groups with respect to:

1. Time to
  - Initiation of renal replacement therapy or
  - Death
2. Neuron-specific enolase (NSE) level at 48 h
3. MOCA score at 3 months (lowest score allocated to patients not available for follow-up, including deceased patients)
4. Modified Ranking Scale (mRS) and CPC at 3 months
5. NT-pro-BNP at 3 months (highest value allocated to patients not available for follow-up)
6. eGFR and LVEF at last available measurement within 3 months of follow-up
7. Daily cumulated vasopressor requirement during ICU stay and need for combination of vasopressors and inotropic agents or mechanical circulatory support

##### Safety

The OHCA population admitted to intensive care is a critically ill group of patients. Most adverse events may be of a potentially serious nature but are often common in this patient group. Cardiac arrest patients and patients being unconscious for any other reason are at great risk of developing adverse events. Adverse events will be recorded daily according to 7.1. With regard to the trial intervention, relevant SAEs are considered to be death, need for mechanical hemodynamic support, and need for renal replacement therapy in the first 3 days.

##### Safety variables-adverse events (AE)

Major bleeding: Uncontrolled bleeding (> 1 unit of blood/10 kg/1 h), bleeding causing fatality; symptomatic bleeding in critical organ, e.g., intracranial, intraspinal, intraocular, intraarticular, pericardial; other bleeding, e.g., retroperitoneal, muscular, solid organ, thoracic with Hb ↓ > 50 g/L (5 g/dl; 3.1 mmol/L) and requiring > 2 units of transfused blood

Infection: Severe sepsis^1^, septic shock^2^, pneumonia during or after ventilator therapy, others

Renal impairment: Need for continuous renal replacement therapy (CRRT) or intermittent hemodialysis (IHD)

Electrolyte disorders: Hypokalemia (< 3.0 mmol/l), hypophosphatemia (< 0.7 mmol/l), hypomagnesaemia (< 0.7 mmol/l)

Metabolic disorders: Sustained hyperglycemia (> 10 mmol/l > 4 h), hypoglycemia (< 3.0 mmol/l)

Arrhythmia: VF, VT, tachycardia > 130/min, bradycardia < 40/min, atrial flutter, atrial fibrillation, need for pacing, circulatory collapse mandating CPR

Seizures: Tonic-clonic, myoclonic, electrographic status epilepticus

### Participant timeline {13}

Safety variables and adverse events will be recorded continuously during the first 7 days, when the patient is in the intensive care unit. Adverse event occurring later than this will be evaluated at the scheduled follow-up visit at 90 days. Hereafter, no follow-up is planned.

See SPIRIT Table 1 and sections describing primary and secondary outcomes.

**Table 1:** Schematic presentation of the study time line

	Study period										
Enrollment	Pre-allocation	Post-allocation									Follow-up
Timepoint	ROSC to randomization	0 h	6 h	12 h	24 h	36 h	48 h	72 h1	96 h1	120 h1	Days 30 and 90
**Enrollment**											
**Eligibility screen**	x										
**Informed consent**		Legal guardian and next of kin									Patient as soon as possible
**Randomization**	x										
**Interventions**											
**Target blood pressure**		x					Until invasive BP monitoring is discontinued				
**Oxygenation**		x					Until extubation				
**Active post-TTM fever management**		x				TTM device discontinued after 36 or 72 h					
**Assessments**											
**Biochemestry2**		x			x		x	x	x	x	
**Troponin T and CKMB**		x	x	x	x	x	x	x			
**Biobank samples**		x			x		x				
**ECG**		x	x	x	x	x	x	x	x	x	
**Swan-Ganz-based measurements**		x	x	x	x	x	x	x			
**Automated pupillometry**		x	x	x	x	x	x	x	x	x	
**ABG and VBG**		x	x	x	x	x	x	x			
**Creatinine clearance**							x		x		
**CPC and mRS score**											x
**MOCA score**											Only day 90

*ROSC* return of spontaneous circulation, *TTM* Targeted Temperature Management, *ECG* electrocardiogram, *ABG* arterial blood gas, *VBG* venous blood gas, *SOFA score* Sequential Organ Failure Assessment, *CPC* Cerebral Performance Category, *mRS* modified Rankin Score, *MOCA* Montreal Cognitive Assessment

1: Assements made if patient is still admitted to the ICU

### Sample size {14}

Sample size estimation is based on blinded BP target allocation and on the assumption that no interaction with the oxygen intervention exists.

The combined primary outcome is time to death or hospital discharge in a state of CPC 3 or 4. We are planning a study with 400 experimental subjects, 400 control subjects, an accrual interval of 48 months, and additional follow-up after the accrual interval of 3 months. Prior data indicate the 6-month mortality is 33% overall. Assuming a mortality of 28% in the superior group compared to 38% in the inferior groups, we will need to include 732 patients in total or 846 patients in total to achieve a power of 0.8 and 0.9, respectively. The type I error probability associated with this test of the null hypothesis that the experimental and control survival curves are equal is 0.05.

Loss of final measurement is expected, but from the experience from the previous trial, the number of missing follow-up assessments is small (< 5%) and will not result in an increase of the number of patients needed.

### Recruitment {15}

Patients are included in the trial when arriving at the tertiary center and if all inclusion criteria and no exclusion criteria are present. The formal inclusion and randomization are performed by the attending physician via a dedicated website.

### Assignment of interventions: allocation

#### Sequence generation {16a}

The allocation of treatment goals has been performed in a 1:1:1:1 manner, using varying block sizes of 4–16.

The randomization to duration of device-based active temperature intervention (substudy) has been performed independently.

#### Concealment mechanism {16b}

Inclusion and randomization are performed via a dedicated website, and the allocation is kept concealed until the time of randomization (oxygenation target and duration of device-based post-TTM temperature management). The blood pressure intervention is concealed as the blood pressure module is labeled by a unique ID number, which is the patient’s study ID. The blood pressure module has been adjusted as described above before the inclusion of the patient.

#### Implementation {16c}

The attending physicians will have access to the trial inclusion webpage and assess eligibility criteria and inclusion of patients in the trial. The webpage will automatically request consent from the assigned.

### Assignment of interventions: blinding

#### Who will be blinded {17a}

Staff and study personnel will remain blinded to the allocated blood pressure target, as information of the blood pressure allocation is available to the core lab, adjusting the blood pressure modules and the person able to perform unblinding if deemed necessary by the treating physician.

#### Procedure for unblinding if needed {17b}

A person will be available for unblinding if necessary. However, as unblinding should only occur for safety reasons, and the blood pressure module may be switched to a clinical non-manipulated device with minimal delay, we expect a limited need for unblinding.

### Data collection and management

#### Plans for assessment and collection of outcome {18a}

Individual patient data will be handled as ordinary chart records and will be kept according to the legislation (e.g., data protection agencies) of the Danish health system. The study uses electronic data collection only, besides the consent forms that are on paper. The study database will be maintained for 15 years and anonymized if requested by the relevant authorities.

Danish legislation regarding the respect for patients’ physical and mental integrity and rights will be respected. Approval for storing data relevant to the trial, including potentially sensitive information, will be sought from the relevant authorities.

#### Plans to promote participant retention and complete follow-up {18b}

Staff dedicated to the ensure participant retention and promote adherence to the planned follow-up visit has been allocated to both sites involved in the trial. When participants are unable to attend an in-hospital follow-up visit, we offer a visit in the patients’ home, a telephone-based assessment of the neurological outcome, and mailing the questionnaires to the patient, in that order. Patients and relatives are not offered financial support for visiting, but expenses for transportation may be covered in selected cases.

Patients who discontinue or deviate from the intervention protocols will remain in the study database, if a consent for the data handling can be obtained, in which case a full data entry will be provided.

#### Data management {19}

Data is stored in a RedCAP® database hosted by the Regional IT department. Data is entered by dedicated study personnel and data quality is monitored. Data quality is monitored by the primary investigator by random sampling. A GCP monitor will review and validate consent forms, eligibility criteria for the trial, and endpoint data.

#### Confidentiality {27}

Confidentiality is ensured by the database system (RedCAP® and that medical data and patient’s sensitive data is accessed by trained medical staff only with valid authorization with the Danish Health Care Authorities). Anonymized data for meta-analyses may be provided after the publication of the main manuscripts.

#### Plans for collection, laboratory evaluation, and storage of biological specimens for genetic or molecular analysis in this trial/future trials {33}

The database may be supplemented by other clinical information and by novel analyses of the biobank material. The ethics committee will be consulted if there is uncertainty of the planned analyses or data catchment is covered by the informed consent

### Statistical methods

#### Statistical methods for primary and secondary outcomes {20a}

The primary outcome (death or severe neurological injury at time of hospital discharge CPC 3–4) for the blood pressure and oxygenation interventions will be analyzed and reported separately in a proportional hazard model (proc phreg) as a crude analysis with no co-factor adjustments. The number and fraction of the population with the endpoint will be reported.

No interaction of the two interventions is expected, and the interaction of the two will be analyzed and reported with the blood pressure target intervention. A Kaplan-Meier estimate plot will be presented with a maximum of 180 days.

Baseline and demographic data will be presented in a table, but no formal statistical testing will be performed due to the randomized design of the trial. A figure presenting the mean blood pressure or PaO_2_ respectively ±1.96SD (representing 95% of the observed cases) will be presented.

For the secondary outcomes, the time to renal replacement therapy and time to death will be compared in the proportional hazard model. NSE, MOCA score, Modified Rankin scale and Cerebral performance score, NT-pro-BNP, eGFR, and LVEF (at 3 months or last known value) and daily cumulated vasopressor requirement during the ICU stay will be reported and compared by a *t* test, and logarithmic transformation will be applied if data is skewed.

Safety variables: Major bleeding, infection, renal impairment (need for dialysis), electrolyte or metabolic disorders, arrhythmia, and occurrence of seizures and or shivering will be reported and compared by the chi-square test.

#### Statistical software

SAS studio, ver 3.7.

#### Interim analyses {21b}

For interim analyses, there will be used an alternative time point of 7 days instead of 90 days for the primary and selected secondary outcomes (death). Interim analysis after inclusion of 400 patients is planned.

#### Methods for additional analyses (e.g., subgroup analyses) {20b}

Predefined subgroup analyses including the following predefined variables will be performed and presented as a forest plot

1. Sex
2. Age above the median age for the population
3. Known renal failure (defined as eGFR < 30 ml/min or current renal placement therapy) at time of cardiac arrest
4. Known hypertension at time of cardiac arrest
5. Known chronic obstructive pulmonary disease at time of cardiac arrest
6. Shockable primary rhythm
7. Suspected STEMI as cause of cardiac arrest
8. Site

#### Methods in analysis to handle protocol non-adherence and any statistical methods to handle missing data {20c}

Trial sites will be asked to complete all CRFs and other forms if missing data is found in the electronic database. Missing data will be reported in the publication. More than 5% missing data will result in multiple imputation with the creation of 5–10 imputed datasets to be analyzed separately and the aggregated into one estimate of intervention effect on the primary and secondary outcomes [59, 60]. Analyses will be performed according to the modified intention to treat principle with patients lost to follow-up included in the denominator.

#### Plan to give access to the full protocol, participant-level data, and statistical code {31c}

Access to the protocol will be given on request. Access to participant-level data will be given only if the data regulation authorities permit.

### Oversight and monitoring

#### Composition of the coordinating center and trial steering committee {5d}

The trial steering committee consists of JK, HS, JEM, and CH. This group represents the two sites participating in the trial. Rigshospitalet is the coordinating center.

The Good Clinical Practice unit at the research department of the Cardiology Research Unit oversees that the study is performed according to the principles of good clinical practice. The GCP report to the study sponsor.

#### Composition of the data monitoring committee, its role, and reporting structure {21a}

The data monitoring committee consists of Professor Lars Køber (Chair), Department of Cardiology, The heart Center Rigshospitalet; Professor Kirsten Møller, Department of Neuroanaesthesiology, The Neuroscience Centre and Institute for Clinical Medicine, Faculty of Health Sciences, University of Copenhagen; and Ass. Professor Jens Jacob Thune, Department of Cardiology, Bispebjerg Hospital.

#### Adverse event reporting and harms {22}

Adverse reporting is performed according to good clinical practice principles. However, since death is frequent, the scientific ethics committee is only informed of the number of deaths and other adverse events annually, unless the adverse are suspected to be directly related to the protocolized interventions.

#### Frequency and plans for auditing trial conduct {23}

Planned interim analyses have been performed after 200 or 400 patients have been included in the trial and the DSMB has given their advice directly to the Steering committee. A charter for the DSMB has been published, but the DSMB will have unlimited access to data if requested.

#### Plans for communicating important protocol amendments to relevant parties (e.g., trial participants, ethical committees) {25}

Protocol revisions have been performed and have been communicated to the ethics committee according to their requirements. No changes in the current protocol are planned. Any deviations from the protocol will be fully documented using a breach report form. No updates to the protocol in the clinical trial registry https://www.clinicaltrials.gov/ct2/show/NCT03141099 are planned, other than updating the trial status.

#### Dissemination plans {31a}

The results of the trial will be published, regardless of being positive, negative, or inconclusive. The result of the two major interventions (blood pressure and oxygenation targets) will be published in two separate scientific papers.

The trial will be analyzed by the steering group (SG) and the results interpreted by the SG with data coded as “A” and “1” and the allocation of the patients will not be revealed until the SG agrees on the statistical analysis and drafts a conclusion based on the primary outcome. A manuscript will be drafted at a closed meeting by a predefined writing group consisting of the SG members. The final manuscript will be submitted to a peer-reviewed international journal. Authorship will be granted using the Vancouver definitions and depending on personal involvement. The author list including steering group members and others who fulfilled the Vancouver criteria as well as the chair of the DSMB will be offered co-authorships. Centers recruiting ≥50 patients will be entitled to one name (additional names) per 50 patients randomized in the trial. A reference to an appendix with all sites, site investigators, and number of patients enrolled will be made.

## Discussion

### Blood pressure intervention

Blood pressure is an easily accessible parameter in describing and monitoring the patients’ hemodynamics and is thus a central part of the monitoring in most patients admitted to critical care. While blood pressure alone does not describe perfusion or oxygen delivery, many consider blood pressure as one of the main goals in managing patients with critical conditions, including post-cardiac arrest [19, 20]. A clinical study assessed the existence of the cerebral autoregulation in post-cardiac arrest victims, by manipulating blood pressure by administration of nor-epinephrine in 18 patients, finding an impaired cerebral autoregulation in 44% of the patients [61]. The same study identified a lower limit of cerebral autoregulation at 114 mmHg (range 80–120 mmHg) in patients with preserved autoregulation, compared to 76 mmHg in normal volunteers, indicating that current practice targets may be sub-optimal [61]. Impaired cerebrovascular autoregulation has also been found to be associated with poor neurological and clinical outcomes in a small study [62]. The clinical consequences of targeting blood pressures below the limits of cerebral autoregulation in the management of post-cardiac arrest patients remain to be resolved.

A systematic review concluded from 9 observational studies that higher blood pressure is associated with improved neurological outcome after cardiac arrest and acknowledging a significant risk of bias, not only due to the observational study designs, but also due to a significant heterogeneity between studies [63]. Blood pressure has been measured as mean arterial pressure (MAP), systolic blood pressure (SBP), or a time-weighted average of MAP (TWA-MAP); outcomes varied for neurological outcome and mortality [63].

Previous studies have reported a significant higher value for MAP during TTM than what seems to be current practice. The Bernard trial of 2002 reported mean MAP values ranging from 88 to 108 during ICU admission [16], whereas a more contemporary trial, the TTM trial, found MAP ranging from 75 to 80 mmHg during TTM [28]. This study found a steep increase in risk of death for MAP below 65 and only a modest protective effect of higher blood pressure values [28]. Furthermore, a consistent association of increasing need for vasopressors and poor neurological outcome or mortality has been reported [28].

Two recent, relatively small clinical open-label prospective trials have investigated two different targets for blood pressure during targeted temperature management since the present trial was initiated. Ameloot et al. compared targeting a MAP of 85–100 to 65 mmHg, finding no difference in the primary outcome of the extent of neurological injury on MRI of the brain and similar fraction of patients with good neurological outcome [22]. A second study by Jakkula et al. compared a low-normal blood pressure of 65–75 mmHg to high-normal blood pressure 80–100 mmHg, finding no differences in NSE levels at 48 h and no differences in the clinical secondary outcomes [23].

The current European Resuscitation Council guidelines suggest a target blood pressure sufficient for maintaining a urine output of 0.5 ml/kg/h and to avoid blood pressure < 65 mmHg [20], whereas the American Heart Association guidelines suggest targeting a MAP greater than 65 mmHg or greater than 90 mmHg systolic (class IIa, LOE B-NR) [19]. As part of a survey distributed among the participating centers in the TTM trial, sites reported a standard blood target varying between MAP of 60 and 75 mmHg (personal communication).

Thus, the current body of evidence supporting MAP targets of 65–75 mmHg is very weak, and a clinical equipoise of optimal target blood pressure in the interval of approximately 65 to 75 mmHg or higher exists.

### Oxygenation intervention

Preclinical data suggest that hyperoxia increases cerebral damage and neurological dysfunction following brain ischemia. Most data originates from cardiac arrest models in which a no-flow state is induced and cerebral injury or neurological dysfunction is assessed [20–22]. Fairly small RCTs in humans have found increased levels of NSE but not S-100B in patients randomized to FiO_2_ = 100% compared to FiO_2_ = 30% [23]. Furthermore, a post-analysis of patients mechanically ventilated after suffering non-traumatic brain injury seems to have a poorer outcome with high oxygenation targets [35].

Hyperoxia during and after ischemia has been investigated in several settings, and a final consensus of its benefits and hazards has yet to be established. In the following, the term hyperoxia is used in situations where the FiO_2_ is increased to levels above 50% for simplicity.

Hyperoxia may increase the risk of developing lung injury (hyperoxia-induced lung injury (HALI)), which carries a high morbidity and mortality. However, this condition seems to require several days of extremely high FiO_2_ to be significant and various ventilation modes may have a significant impact as well [24]. The risk of HALI associated with shorter periods of hyperoxia seems small.

Ischemia-reperfusion injury has been investigated in smaller studies only in relation to circulatory bypass with no significant impact on the magnitude of increase in biomarkers related to cardiac injury according to a review by [25]. Oxygen administration during transport for primary PCI in patients with ST segment myocardial infarction has recently been associated with increased release of creatinine kinases and larger final infarcts size on cardiac magnetic resonance imaging [26]. The impact of shorter courses of oxygen supplement during weaning from circulatory bypass has yet to be investigated. A direct influence of hyperoxia with an increase in systemic vascular resistance has also been suggested, and hyperoxia may not be associated with improved tissue oxygenation [27], while the clinical effects of this finding remain to be understood [25].

A recent systematic review and meta-analysis suggested that the risk of surgical site infections may be reduced with hyperoxia [28]. However, a larger RCT in abdominal surgery found an increased mortality with hyperoxia, in particular in patients undergoing surgery for malignancies [29].

In summary, hyperoxia may reduce risks associated with surgery and critical illnesses with regard to surgical site infections and possibly reperfusion injury, whereas the effects on overall benefit in terms of mortality may be detrimental. Further research is needed, and for the time being equipoise with regard to the benefits of different targets for oxygenation. Most patients will require some degree of oxygen supplement when lungs are ventilated after OHCA; hence, this study pragmatically studies low normal, i.e., “restrictive” PaO_2_ of 9–10 kPa (target 9.5 kPa), and high normal, i.e., “liberal” PaO_2_ of 13–14 kPa (target 13.5 mmHg). FiO_2_, PEEP, and lung recruitment maneuvers for maintaining the assigned PaO_2_ are left at the discretion of the treating physician.

## Trial status

Protocol version 3.2 (07JAN2018).

Recruiting; the first patient was included on March 10, 2017. The last patient was included on December 11, 2021. Completion of the 90-day follow-up before May 2022 and unblinding and statistical analysis will begin hereafter.

## Abbreviations

CA: Cardiac arrest
CPC: Cerebral Performance Category
CVP: Central venous pressure
eGFR: Estimated glomerular filtration rate
GCS: Glasgow Coma Scale
ICU: Intensive care unit
LAP: Left atrial pressure
LOE: Level of evidence
LVEF: Left ventricular ejection fraction
MAP: Mean arterial pressure
MoCA: Montreal Cognitive Assessment
MRI: Magnetic resonance imaging
mRS: Modified Rankin Scale
NSE: Neuron-specific enolase
OHCA: Out-of-hospital cardiac arrest
PAP: Pulmonary artery pressure
PCI: Percutaneous coronary intervention
PEEP: Peak end-expiratory pressure
Pro-BNP: Pro-brain natriuretic peptide
ROSC: Return of spontaneous circulation
SBP: Systolic blood pressure
TTM: Targeted Temperature Management

## Footnotes

1 Sepsis: Infection is the inflammatory response to the presence of microorganisms or the invasion of normally sterile host tissue by those organisms. The Systemic Inflammatory Response Syndrome (SIRS) is diagnosed when the patient exhibits two of the following four abnormalities:

- Temperature > 38 °C or < 36 °C
- Heart rate > 90 beats min^−1^
- Respiratory rate > 20 breaths min^−1^ or PaCO2 < 4.3 kPa or need for mechanical ventilation
- White blood cell count > 12,000 cells mm^−3^ or < 4000 cells mm^−3^ or > 10% immature cells (band forms)

Sepsis is SIRS resulting from infection. Severe sepsis is sepsis associated with organ dysfunction, hypoperfusion or hypotension. Hypoperfusion and perfusion abnormalities may include, but are not limited to, lactic acidosis, oliguria, or an acute alteration in mental status.

2 Septic shock is sepsis with hypotension (systolic blood pressure < 90 mmHg or a reduction of > 40 mmHg frombaseline) and perfusion abnormalities (see severe sepsis above) or the requirement for vasoactive drugs despite adequate fluid resuscitation in the absence of other causes for hypotension.
